# Classification and Monitoring of Salt Marsh Vegetation in the Yellow River Delta Based on Multi-Source Remote Sensing Data Fusion

**DOI:** 10.3390/s25020529

**Published:** 2025-01-17

**Authors:** Ran Xu, Yanguo Fan, Bowen Fan, Guangyue Feng, Ruotong Li

**Affiliations:** 1 School of Oceanography and Spatial Information, China University of Petroleum East China—Qingdao Campus, Qingdao 266580, China; s22160019@s.upc.edu.cn (R.X.); z21160108@s.upc.edu.cn (G.F.); s23160011@s.upc.edu.cn (R.L.); 2 College of Underwater Acoustic Engineering, Harbin Engineering University, Harbin 150001, China; fanbowen@hrbeu.edu.cn

**Keywords:** salt marsh vegetation, Yellow River Delta, remote sensing, SAR polarization, vegetation classification

## Abstract

Salt marsh vegetation in the Yellow River Delta, including *Phragmites australis* (*P. australis*), *Suaeda salsa* (*S. salsa*), and *Tamarix chinensis* (*T. chinensis*), is essential for the stability of wetland ecosystems. In recent years, salt marsh vegetation has experienced severe degradation, which is primarily due to invasive species and human activities. Therefore, the accurate monitoring of the spatial distribution of these vegetation types is critical for the ecological protection and restoration of the Yellow River Delta. This study proposes a multi-source remote sensing data fusion method based on Sentinel-1 and Sentinel-2 imagery, integrating the temporal characteristics of optical and SAR (synthetic aperture radar) data for the classification mapping of salt marsh vegetation in the Yellow River Delta. Phenological and polarization features were extracted to capture vegetation characteristics. A random forest algorithm was then applied to evaluate the impact of different feature combinations on classification accuracy. Combining optical and SAR time-series data significantly enhanced classification accuracy, particularly in differentiating *P. australis*, *S. salsa*, and *T. chinensis*. The integration of phenological features, polarization ratio, and polarization difference achieved a classification accuracy of 93.51% with a Kappa coefficient of 0.917, outperforming the use of individual data sources.

## 1. Introduction

Salt marshes are extensive coastal intertidal zones situated between terrestrial areas and open water that are frequently inundated by tides [1]. They provide a range of ecological functions, such as habitat provision and carbon sequestration [2]. However, due to climate change and anthropogenic disturbances, salt marshes are facing severe degradation [3,4]. The remote sensing of wetlands can address the limitations of traditional field surveys, enhancing efficiency [5]. Phenology refers to the seasonal phenomena exhibited by vegetation in response to climatic conditions, such as budding, leafing, flowering, fruiting, and senescence. Analyzing significant changes in vegetation time series and extracting key time points and characteristic variables is crucial for understanding these processes [6]. Li Jialin [7] analyzed the intra-annual variation of vegetation indices in the Spartina alterniflora salt marshes along the Jiangsu coast, determining the growth status by distinguishing between growing and non-growing seasons. Davranche et al. [8] extracted 17 remote sensing indices using multi-seasonal SPOT-5 monthly imagery, and they combined these with vegetation phenological parameters to classify and map vegetation in the Camargue wetlands in France using decision tree classification. However, optical remote sensing is highly susceptible to weather conditions, making it unsuitable for the continuous monitoring and analysis of salt marsh vegetation [9].

SAR is unaffected by weather conditions and has strong penetrative capabilities, which help address the limitations of optical remote sensing, such as susceptibility to cloud cover and rainy weather, and insensitivity to moisture information [10]. SAR is advantageous for effectively extracting wetland information [11]. Tsyganskaya et al. [12] used time-series data from Sentinel-1, combining pixel-based and object-based approaches to monitor temporarily flooded vegetation, improving the accuracy of flood extent extraction. Similarly, Martinez and Toan (2006) [13] employed SAR imagery from Envisat ASAR (HV and HH bands) and ERS-2 (VV band) to map wetland dynamics in the Paraná River wetlands of Argentina. Thus, time-series SAR data hold great potential for wetland classification and monitoring [14]. With global coverage, free access, and a 12/6-day revisit cycle, Sentinel-1 SAR is highly suitable for temporally dense wetland classification and monitoring [15]. Fully utilizing the characteristics of wetlands in SAR imagery is key to accurate classification and effective monitoring. In current studies on the distribution of wetland vegetation in the Yellow River Delta, most research relies on single-temporal imagery as the primary data source with the majority using single optical imagery for vegetation classification and identification. These studies typically utilize vegetation indices, texture features, and spectral characteristics as classification features for wetland vegetation. However, such methods are inadequate for the dynamic monitoring of wetland vegetation, refs. [16,17,18] and the classification accuracy requires significant improvement. Moreover, research specifically targeting *T. chinensis* in the Yellow River Delta remains limited. In most cases, *T. chinensis* and reeds are grouped together without proper differentiation, resulting in a lack of detailed classification.

Effectively combining the strengths of optical and SAR data allows for the extraction of useful information, enhancing the ability to identify land cover features. Erinjery et al. [19] integrated Sentinel-1 SAR data with Sentinel-2 optical data, significantly improving the accuracy of vegetation type extraction in the Western Ghats. Similarly, Li et al. [20] used radar remote sensing imagery to classify mangrove communities and estimate biomass, overcoming the limitations of using optical remote sensing alone, thus providing essential information and new monitoring methods for mangrove wetland conservation. Veloso et al. [21] monitored crop temporal trajectories using both Sentinel-1 SAR data and optical imagery, highlighting the importance of SAR data, particularly the VH/VV bands. The classification of salt marsh wetlands using multi-feature approaches is becoming a major trend in wetland research and development with the integration of Sentinel-1 radar data and Sentinel-2 optical data providing efficient and reliable technological solutions for ecological conservation and agricultural management. Mashaba-Munghemezulu et al. [22] combined Sentinel-1 and Sentinel-2 data with random forest (RF), support vector machine (SVM), and stacked model (ST) algorithms to precisely delineate smallholder maize farms in South Africa. Their results demonstrated that the fusion of multi-source data significantly improved classification accuracy, offering strong support for food security monitoring. Similarly, Sun et al. [23] utilized time-series Sentinel-1 and Sentinel-2 imagery and applied a recursive feature selection method to optimize SAR and optical feature combinations. Their study achieved an overall accuracy of 83.22% in crop-type mapping for oasis agricultural areas in Xinjiang, highlighting the potential of red-edge bands in improving crop classification accuracy. In the domain of wetland vegetation and boundary monitoring, Ruiz et al. [24] employed Sentinel-1 and Sentinel-2 data combined with Geographic Object-Based Image Analysis (GEOBIA) to classify vegetation species in subtropical wetlands. Their approach achieved a classification accuracy of 91.3% using RF, underscoring the critical role of SAR features in differentiating wetland vegetation types. Yang et al. [25] proposed a novel method to extract tidal flat boundaries by integrating radar data acquired during low tide with optical data acquired during high tide. Using new spectral indices and a simple thresholding approach, their study significantly enhanced the efficiency and accuracy of tidal flat monitoring, providing robust technical support for the conservation of coastal wetland ecosystems. Moreover, Yu et al. [26] integrated Sentinel-1 and Sentinel-2 imagery with terrain data to assess object-based and pixel-based classification methods for wetland information extraction. Their results demonstrated that object-based segmentation performed better in mountainous and hilly wetland areas, achieving classification accuracies of 96.00% and 96.67% for water bodies and built-up land, respectively. Cherian et al. [27] utilized optimized feature selection techniques, such as Jeffries–Matusita distance and Spearman correlation analysis, to combine SAR and optical data for the characterization of coastal wetlands using RF and SVM classifiers. Their findings indicated that data fusion improved classification accuracy by 33%, validating the complementarity of radar and optical data in wetland monitoring. These studies collectively highlight the indispensable role of radar data in all-weather observations and vegetation structure analysis as well as the distinct advantages of optical data in extracting spectral information.

Previous studies have attempted to combine optical and SAR data for vegetation classification; most have remained at the level of simple feature stacking, lacking a deep integration of optical phenological features and SAR polarization characteristics. Building on this foundation, this study introduces a novel approach by extracting annual phenological features from optical data—such as the start and end of the growing season, maximum NDVI values, and seasonal variation trends—and integrating them with SAR polarization features, including polarization ratios and differences. This organic fusion of optical time-series information and SAR structural characteristics enhances the ability to capture vegetation dynamics and structural attributes. Moreover, it significantly improves the differentiation of similar vegetation types, such as *P. australis* and *T. chinensis*, in complex wetland environments.

## 2. Materials

### 2.1. Study Area

**Figure 1:**
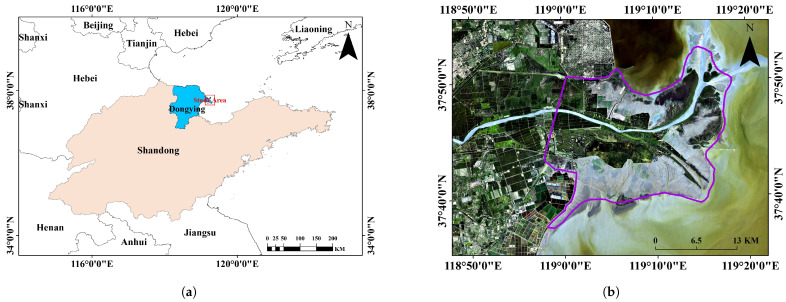
Location of the study area. (**a**) Approximate location of the study area. (**b**) The purple outlined area represents the specific study area.

The Yellow River Delta, located in Dongying City, Shandong Province, is the largest newly formed delta along China’s coast [28]. It is home to the Yellow River Delta National Nature Reserve, established in 1992, with 55.1% natural vegetation coverage, making it the largest wetland vegetation area in coastal China [29]. The region experiences a temperate climate with distinct seasons and consists of simple plant communities, including *P. australis*, *Cyperus* spp., *S. salsa*, *Spartina alterniflora*, and *T. chinensis*. *P. australis* dominates freshwater and low-salinity areas, forming dense communities, while in highly saline soils, its growth is stunted. *T. chinensis* is prevalent in salt marshes and tolerates high salinity, often coexisting with *S. salsa*. *S. salsa*, a key salt-tolerant species, thrives in coastal mudflats and highly salinized regions, forming dense communities in moderate salinity, but its coverage declines in extreme conditions. These species reflect the delta’s dynamic, salinity-driven ecosystem.(Shandong Yellow River Delta National Nature Reserve Administration, http://hhsjzzrbhq.dongying.gov.cn, accessed on 2 September 2018). In this study, a portion of the Yellow River Delta was delineated as the research area (Figure 1).

### 2.2. Remote Sensing Data and Preprocessing

Sentinel-1 is a C-band synthetic aperture radar (SAR) dataset. The selected data are in ground range detected (GRD) format with dual polarization, featuring a spatial resolution of 10 m and polarization modes of VV and VH. The preprocessing of Sentinel-1 data was conducted using the SNAP platform. The preprocessing steps include (1) orbit correction; (2) noise removal; (3) radiometric calibration; (4) image filtering using the Lee Sigma filter; (5) terrain correction; and (6) conversion to decibels. After preprocessing, polarization ratio and phase difference calculations were performed, which were followed by the computation of average values. The Sentinel-2 constellation consists of two identical satellites operating in a sun-synchronous orbit, allowing for a 5-day revisit at the equator with even shorter revisit times at the poles [30]. Sentinel-2 MSI reflected radiation across 13 spectral bands, ranging from visible and near-infrared to shortwave infrared. The spatial resolution is divided into 10 m, 20 m, and 60 m. In this study, 10 m and 20 m resolution data were selected. The data were downloaded from the European Space Agency (ESA) and had undergone radiometric correction. Atmospheric correction was performed using Sen2Cor to generate Level 2A data. In SNAP, the 20 m data were resampled to 10 m. Pixels with values of 3 and 8–11 were masked using the scene classification map. After cloud masking, the images were arranged in chronological order to form a time series (Table 1).

### 2.3. Sample Data and Validation Data

The sample data and validation data consist of three components. (1) Field surveys: In July 2020, a field survey was conducted in the study area. To ensure the purity of the samples, plots with a single vegetation coverage greater than 20 m × 20 m were selected. The survey results indicated the presence of three dominant species in the study area: *S. salsa*, *P. australis*, and *T. chinensis*. Most of the surveyed plots were located near roads and dikes. (2) Unmanned Aerial Vehicle (UAV) Survey: Simultaneously with the field investigation, UAV-based surveys were carried out. A DJI UAV was deployed to capture aerial images of representative vegetation areas in the Yellow River Delta. The surveys were conducted under clear weather conditions with moderate wind speeds, and flight altitudes were maintained between 100 m and 150 m. The UAV data were processed using software to generate orthophotos by synthesizing red, green, and blue (RGB) spectral bands. (3) Supplementation with Google Earth and High-Resolution Optical Imagery: Due to the presence of extensive marshes and mudflats in the Yellow River Delta, certain areas were inaccessible to both human investigators and UAVs. To address this limitation, high-spatial-resolution images from Google Earth and optical satellite imagery were used to supplement the dataset. UAV imagery and field samples were georeferenced onto Google Earth, and additional samples were interpreted based on the shape, texture, and color of specific vegetation types.

**Figure 2:**
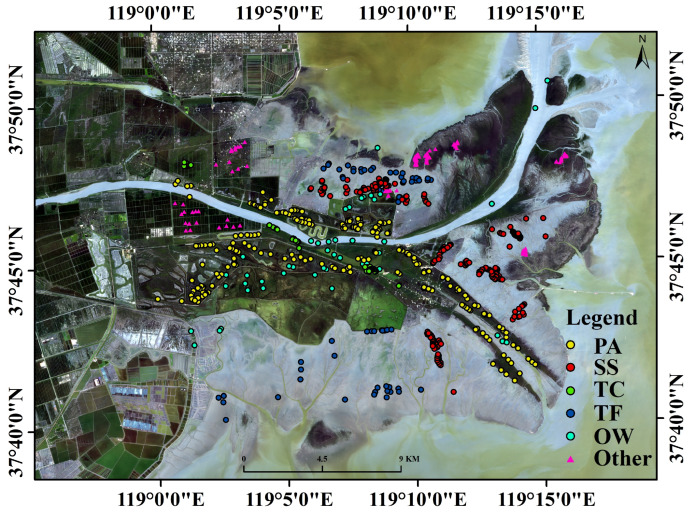
Field data.

This multi-source approach ensured comprehensive sample collection and an accurate representation of vegetation patterns in the study area. Figure 2 illustrates the distribution of samples for each category.

## 3. Methods

### 3.1. Feature Description

Phenological characteristics represent the biological activities of vegetation during different growth stages throughout the year, reflecting growth, development, and senescence. SAR polarization characteristics provide insights into vegetation geometry and surface roughness. VV polarization is sensitive to vertical structures, while VH polarization captures more complex structural details. The VV/VH ratio can reveal differences in vegetation height, density, and coverage.

### 3.2. Tidal Filter

The tidal filtering method proposed in [31] was adopted. The Modified Normalized Difference Water Index (MNDWI) was calculated for each Sentinel-2 image, and a tidal filter was constructed and applied to the NDVI of each image to remove tidal effects. For each pixel, all observations were excluded if the inundation frequency exceeded 0.66. After applying the tidal filter, the remaining pixel data were used to extract parameters through a fitting model.

(1) $$\mathrm{MNDWI}=\frac{\rho_{\mathrm{Green}}-\rho_{\mathrm{SWIR}1}}{\rho_{\mathrm{Green}}+\rho_{\mathrm{SWIR}1}}$$

where $\rho_{\mathrm{Green}}$ and $\rho_{\mathrm{SWIR}1}$ are the surface reflectance values of the green band and short-wave infrared band, respectively.

The filtering criteria are as follows:

(2) MNDWI>−0.1andFreq(MNDWI>−0.1)≤0.66

### 3.3. Fitting Model

In this study, a double logistic (DL) function was employed to fit the time series of pixels after tidal filtering.

### 3.4. Double Logistic (DL)


(3) $$g\left(t,x_{1},\ldots ,x_{4}\right)=\frac{1}{1+\exp\left(\frac{x_{1}-t}{x_{2}}\right)}-\frac{1}{1+\exp\left(\frac{x_{3}-t}{x_{4}}\right)}$$

Here, the basis function is a double logistic function [32]. $x_{1}$ determines the position of the left inflection point, while $x_{2}$ gives the rate of change. Similarly, $x_{3}$ determines the position of the right inflection point, while $x_{4}$ gives the rate of change at this point. Also, for this function, the parameters are restricted in range to ensure a smooth shape.

### 3.5. Feature Extraction

Due to cloud interference, the availability of optical imagery is limited. Therefore, in this study, we integrated preprocessed SAR and optical data to extract phenological and radar polarization features based on the time series of both datasets. The normalized difference vegetation index (NDVI) was calculated using the red (B4) and near-infrared (B8a) bands, and the results were arranged in chronological order. The NDVI was chosen because it is widely used in monitoring vegetation phenology and has demonstrated good accuracy [33,34,35]. Due to the introduction of noise from irregular tidal movements, this study followed recommendations from previous research and applied a tidal filter to all images [31]. After tidal filtering, six commonly used phenological indices were extracted from the pixel-based time series using a double logistic curve fitting method. These indices included two value-based parameters: baseline value (BV) and maximum value (MV); two time-related parameters: start of season (SOS) and end of season (EOS); and two rate-based indices: rate of increase (ROI) and rate of decrease (ROD). The start and end of the season were tested at increments of 10% from 10% to 90%, with 50% being selected as the optimal threshold, as it matched well with field observations of salt marsh vegetation and was consistent with the previous literature [31]. Table 2 provides detailed calculations for these values. Radar polarization features were extracted from Sentinel-1 SAR data, where the backscatter intensity is closely related to the surface roughness and geometric characteristics of ground objects, containing rich spatial information [36]. This study utilized 44 Sentinel-1 SAR images throughout the year to extract the VV and VH polarized backscatter coefficients. The VV-VH polarization difference and the VH/VV polarization ratio were then calculated. For each month, the average value of the polarization ratios and differences from all images was computed, resulting in 12 polarization ratio features and 12 polarization difference features, representing the entire year.

### 3.6. Experimental Design

This study conducted five sets of experiments to classify phenological features, radar features, and combined features. Three groups of classification features were used, and each group was classified using the random forest algorithm, which was followed by an accuracy assessment. To explore the complementary effects of different classification features, these three types of features were combined and classified using random forest with the results tested accordingly. The experiments using only the polarization ratio or polarization difference from radar features alone were excluded from this study due to their poor classification performance in preliminary tests. The combined experiments designed in this study include the combination of phenological features with polarization ratios, phenological features with polarization differences, and phenological features combined with both radar feature types. Finally, the classification accuracy of the five experiments was compared, and the optimal feature combination was selected for classification mapping. The detailed experimental design is presented in Table 3.

### 3.7. Random Forest Algorithm

The relevant features were input into the random forest algorithm to extract salt marsh vegetation. The basic unit of the random forest method is the decision tree, and through ensemble learning, multiple trees are aggregated for classification or regression tasks. The core idea of the random forest method is bootstrap sampling, where multiple sub-datasets are generated by repeated sampling with replacement, and the out-of-bag samples (those not selected in a given bootstrap sample) are used to predict and evaluate the error. After sampling, a decision tree is constructed from each bootstrap sample, and the results from all trees are aggregated. When a new input sample is introduced, each decision tree makes a prediction, and the final output of the random forest is determined by a majority vote, essentially using multiple decision classifiers to determine the final classification result [37]. Two key parameters that affect the output of the random forest algorithm are the number of decision trees and the number of features used by each tree. In this study, the key parameters of the random forest algorithm included the number of decision trees, maximum depth, and the selection of split nodes. Through hyperparameter tuning, we set the number of decision trees to 500 and experimented with different feature selection methods, such as the square root and logarithmic feature selection methods. To avoid overfitting, cross-validation was used to evaluate the model’s generalization performance, and grid search was employed to determine the optimal parameters [38].

### 3.8. Accuracy Evaluation Metrics

The confusion matrix, also known as the error matrix, is the standard format for accuracy assessment. In the evaluation of image classification accuracy, it is primarily used to compare classification results with actual observations. In this study, commonly used metrics including producer’s accuracy (*PA*), user’s accuracy (*UA*), overall accuracy (*OA*), and the Kappa coefficient were selected as evaluation indicators to assess the classification results of different feature combinations. *OA* is the ratio of correctly classified pixels to the total number of pixels. *PA* is the ratio of correctly classified pixels to the actual number of pixels in a given class, while *UA* is the ratio of correctly classified pixels to the total number of pixels classified in that category.

(4) $$PA_{i}=\frac{n_{ii}}{\sum_{j=1}^{r}n_{ij}}$$

Producer’s accuracy (*PA*) measures the proportion of correctly classified samples of a given actual class.

(5) $$UA_{i}=\frac{n_{ii}}{\sum_{j=1}^{r}n_{ji}}$$

User’s accuracy (*UA*) evaluates the reliability of predictions for a given class.

(6) $$\kappa =\frac{p_{o}-p_{e}}{1-p_{e}}$$

(7) $$p_{o}=\frac{\sum_{i=1}^{r}n_{ii}}{N}$$

(8) $$p_{e}=\frac{\sum_{i=1}^{r}\left(n_{i+}\cdot n_{+i}\right)}{N^{2}}$$

The Kappa coefficient (κ) evaluates the classification model’s overall consistency while accounting for chance agreement.

## 4. Results

### 4.1. Analysis of Remote Sensing Phenological Features

**Figure 3:**
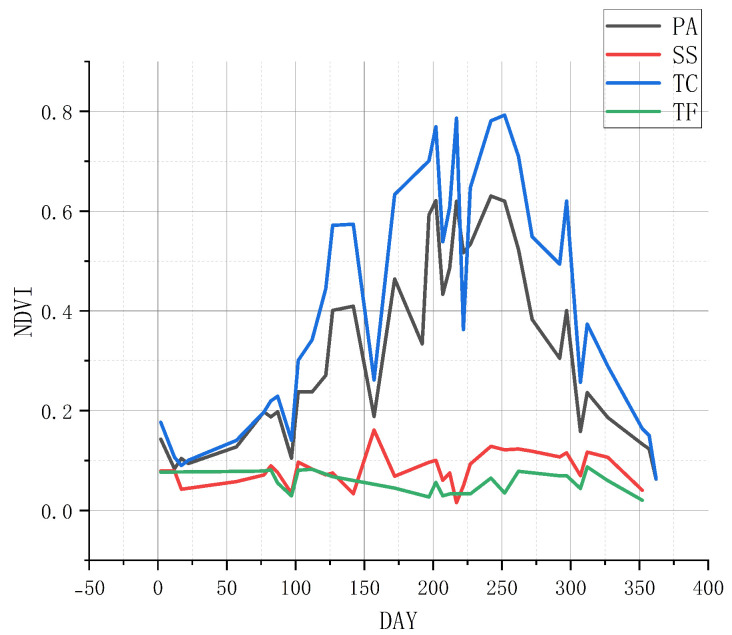
Mean time series variation of sample points. PA = *P. australis*, SS = *S. salsa*, TC = *T. chinensis*, TF = tidal flat.

Figure 3 illustrates the fluctuations and trends in the NDVI time series of the three dominant vegetation types in the study area, reflecting changes in mean NDVI values. Generally, the time series reveal varying degrees of changes among different salt marsh vegetation types, which serves as a basis for vegetation classification. Notably, the curves for *S. salsa* and tidal flats exhibit significant similarity, whereas *S. salsa* shows marked differences compared to *T. chinensis* and *P. australis*. Overall, the variation trends of the three vegetation types follow a unimodal pattern. However, despite the application of tidal filtering, some “noise” remains in the data.

**Figure 4:**
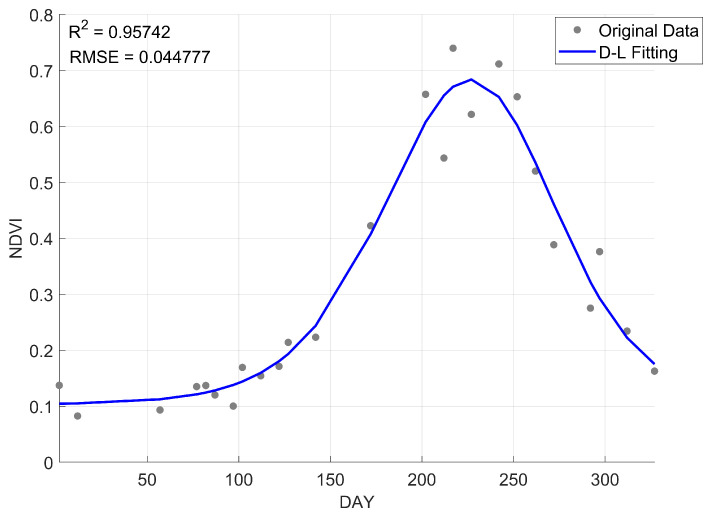
Fitted curve for *P. australis*.

Figure 4 presents the fitted NDVI curve for *P. australis*. After data fitting and reconstruction, the trend becomes more pronounced, allowing for a more accurate representation of the phenological dynamics of wetland vegetation.

**Figure 5:**
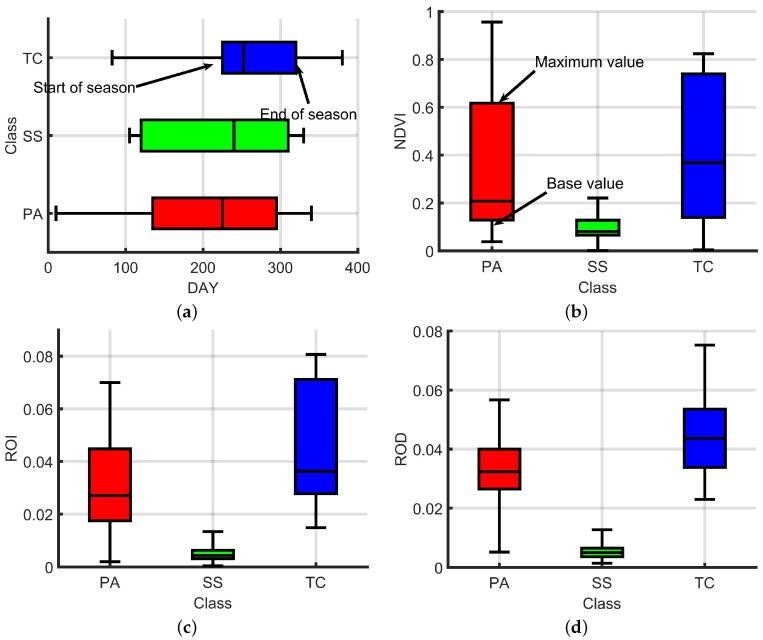
Box plot of phenological features for representative vegetation. Figure (**a**) shows the distribution of the seasonal start and end times for three typical vegetation types. The vertical axes of (**b**–**d**) are unitless, as NDVI, ROI, and ROD are all dimensionless. PA = *P. australis*, SS = *S. salsa*, TC = *T. chinensis*.

Figure 5 illustrates the distribution of phenological parameters for different vegetation types, including the start of the growing season (SOS), the end of the growing season (EOS), the maximum NDVI value (MV), the rate of increase (ROI), and the rate of decrease (ROD). The phenological characteristics of each vegetation type exhibit significant differences, which can be effectively used to distinguish among them. For instance, Figure 5a shows that *P. australis* exhibits an earlier SOS, typically occurring around day 150 (mid-April), which is 20 to 40 days earlier than the other vegetation types. This characteristic reflects the sensitivity of *P. australis* to rising spring temperatures and its adaptive strategy to capture resources early in the growing season. In terms of EOS, *P. australis* usually ends its growing season around day 300 (mid-October), giving it the longest growing season among the vegetation types. This extended period demonstrates its higher resource utilization efficiency and ecological competitiveness. By contrast, Figure 5a shows that *T. chinensis* has a more concentrated and relatively delayed growing season with SOS occurring around day 180 (mid-May) and EOS extending to approximately day 320 (mid-November). This concentrated growing pattern indicates the high efficiency of *T. chinensis* in adapting to environmental changes, especially in utilizing light and water resources in tidal wetland environments. On the other hand, *S. salsa* demonstrates the latest SOS (around day 200, early June) and ends its growing season around day 260 (early September), resulting in the shortest growing season. This phenomenon may be attributed to the salt tolerance and sparse vegetation structure of *S. salsa* with its ecological strategy favoring survival in low-resource-demand environments.

Additionally, regarding the maximum NDVI value (MV), Figure 5b indicates that *T. chinensis* reaches the highest MV during the peak growing season (approximately 0.8), reflecting its dense vegetation canopy and superior photosynthetic capacity compared to the other vegetation types. The MV of *P. australis* is slightly lower than that of *T. chinensis* but significantly higher than that of *S. salsa*. The latter exhibits the lowest MV among all vegetation types (approximately 0.2), indicating its sparse canopy coverage. Regarding the rate of increase in NDVI (ROI), Figure 5c shows that *P. australis* experiences a rapid growth phase, reflecting its strong ability to accumulate resources early in the growing season. In contrast, *T. chinensis* displays a moderate ROI, reflecting a more balanced growth strategy, while *S. salsa* has the lowest ROI, further emphasizing its slow growth dynamics. Similarly, Figure 5d illustrates the rate of decrease in NDVI (ROD) with *P. austral* is exhibiting the fastest decline. This rapid decline indicates a sharp senescence phase at the end of its growing season, where leaf aging and photosynthesis decrease rapidly. In contrast, *T. chinensis* has a slower ROD, suggesting a prolonged senescence phase likely associated with its persistent leaves and vegetation remnants. *S. salsa* has the slowest ROD, which is consistent with its overall slow growth dynamics and lower resource utilization efficiency. The phenological differences among salt marsh vegetation serve as a fundamental basis for vegetation classification.

### 4.2. Analysis of SAR Data Features

**Figure 6:**
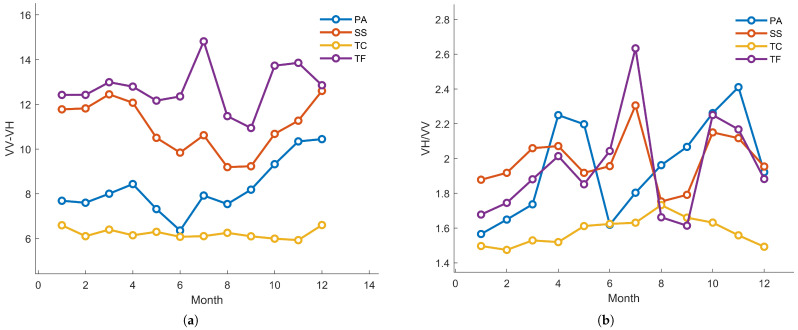
(**a**) Polarization difference time series. PA = *P. australis*, SS = *S. salsa*, TC = *T. chinensis*. (**b**) Polarization ratio time series. PA = *P. australis*, SS = *S. salsa*, TC = *T. chinensis*.

Figure 6 illustrates the polarization characteristics of different vegetation types, including the polarization difference (VV-VH) time series (Figure 6a) and the polarization ratio (VV/VH) time series (Figure 6b). These polarization features exhibit significant differences among the vegetation types, providing an effective basis for distinguishing vegetation types within the study area. For example, Figure 6a shows that the polarization difference of *P. australis* and *T. chinensis* is consistently higher throughout the year compared to that of *S. salsa* and tidal flats. This phenomenon reflects the greater vegetation height and density of *P. australis* and *T. chinensis*, whose complex canopy structures effectively scatter and reflect microwave signals. The polarization difference of *P. australis* shows relatively stable variations but increases slightly during the late growing season (August to October), which may be associated with the accumulation of biomass prior to the end of the growing season. *T. chinensis* exhibits a peak in polarization difference in June, corresponding to the peak biomass during its growing season, indicating that its vigorous growth has a significant impact on microwave scattering. In contrast, *S. salsa* and tidal flats maintain low polarization difference values throughout the year, indicating sparse vegetation coverage and weaker reflected microwave signals. The simple vegetation structure of *S. salsa* and the bare surface of tidal flats further contribute to these low polarization signals.

In terms of the polarization ratio (VV/VH), Figure 6b shows that the polarization ratio of *P. australis* remains consistently high and stable throughout the year. This indicates that its complex vegetation structure enables stable microwave signal scattering, maintaining a high level of reflectance even outside the growing season. The polarization ratio of *T. chinensis* increases significantly from June to August, displaying a pronounced peak, which aligns with its rapid growth in vegetation coverage and biomass during this period. This characteristic suggests that the stronger microwave scattering observed during the growing season is likely a result of its dense vegetation coverage and complex canopy structure. In contrast, the polarization ratio of *S. salsa* exhibits minimal fluctuations and remains at a generally low level throughout the year, reflecting its limited vegetation coverage and reduced microwave reflectance. Tidal flats show the lowest and least variable polarization ratio, indicating the absence of vegetation coverage and the lack of surface structural complexity, resulting in predominantly direct microwave reflection.

In summary, the three typical salt marsh vegetation types exhibit significant differences in polarization difference and polarization ratio characteristics, providing a crucial basis for vegetation classification. The higher polarization difference and polarization ratio values observed in *P. australis* and *T. chinensis* highlight the substantial influence of their complex canopy structures on microwave scattering. In contrast, *S. salsa* and tidal flats, with their simpler structures and sparse coverage, display lower polarization characteristics. These polarization features can not only be used independently for vegetation classification but also integrated with optical phenological characteristics for joint analysis, further enhancing the differentiation of various vegetation types.

### 4.3. Classification Results Analysis

In the process of training a random forest model using different feature sets, five-fold cross-validation was applied to train the model on each training subset and evaluate its performance. The model’s generalization performance was assessed across four dimensions: cross-validation, precision, recall, and F1-score. As shown in Table 4, with the optimization of the feature set and an increase in the number of features, the model demonstrated improved stability and generalization performance. Plan 5 exhibited the highest generalization ability and the best stability.

**Figure 7:**
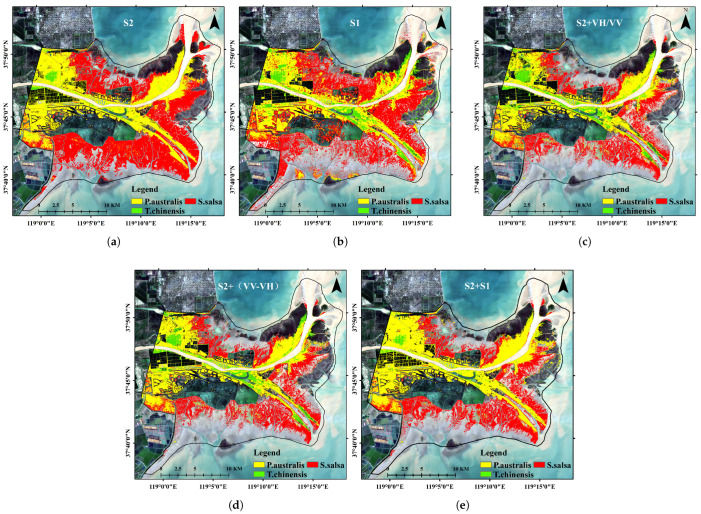
Extraction results of typical salt marsh vegetation. (**a**) Classification results based on optical phenological characteristics; (**b**) classification results based on SAR polarization characteristics; (**c**) classification results integrating optical phenological characteristics and polarization ratio features; (**d**) classification results integrating optical phenological characteristics and polarization difference features; (**e**) classification results integrating optical phenological characteristics and polarization features.

Figure 7 illustrates the classification results of typical salt marsh vegetation, employing five different approaches: based on optical phenological characteristics (Figure 7a), SAR polarization features (Figure 7b), the integration of optical data and SAR polarization ratios (VH/VV) (Figure 7c), the integration of optical data and SAR polarization differences (VV-VH) (Figure 7d), and the comprehensive integration of optical data with both SAR polarization ratios and differences (Figure 7e). The classification accuracy improves progressively with the integration of additional data features, as evident from the visual comparison in Figure 7. This trend is further supported by the classification accuracy statistics summarized in Table 5.

The classification results based on optical phenological characteristics (Figure 7a) exhibit notable limitations with an overall accuracy of only 77.93% and a Kappa coefficient of 0.7288. Although optical data offer certain advantages in spatial resolution, the spectral similarity between vegetation types, particularly the spectral overlap between *P. australis* and *T. chinensis*, leads to significant confusion in the classification results. The user and producer accuracies for this approach are 84.67% and 81.81%, respectively, indicating a substantial level of uncertainty in the classification outcomes.

The classification based on SAR polarization features (Figure 7b) incorporates structural and textural information, resulting in an improved overall accuracy of 80.83% and a Kappa coefficient of 0.8088. Notably, the classification accuracy for *S. salsa* shows significant improvement with a user accuracy of 83.13% and a producer accuracy of 81.94%. However, confusion between *P. australis* and *T. chinensis* persists, indicating that SAR data alone are insufficient to fully distinguish vegetation types with similar structural and spectral characteristics.

By integrating SAR polarization ratios (VH/VV) with optical data (Figure 7c), the classification results showed a further improvement, achieving an overall accuracy of 85.75% and a Kappa coefficient of 0.8791. This approach significantly enhanced the separation between *P. australis* and *T. chinensis* while also providing clearer classification boundaries for *S. salsa*. However, some overlapping regions remain unresolved. The user and producer accuracies reached 91.27% and 89.98%, respectively, reflecting the improved performance of this combined approach. With the incorporation of SAR polarization differences (VV-VH) (Figure 7d), the overall classification accuracy improved to 90.27% with a Kappa coefficient of 0.8977. This approach effectively captured subtle variations in vegetation density and structure, resulting in clearer boundaries among the three typical vegetation types, particularly in mixed areas where classification performance showed significant improvement. The user and producer accuracies for *P. australis*, *T. chinensis*, and *S. salsa* all exceeded 90%, highlighting the advantages of integrating multi-source features.

Finally, through the comprehensive integration of Sentinel-2 optical data with SAR polarization ratios and differences (Figure 7e), the classification achieved the highest accuracy with an overall accuracy of 93.51% and a Kappa coefficient of 0.9172. This approach not only significantly reduced the confusion between *P. australis* and *T. chinensis* but also accurately delineated the distribution of *S. salsa*. The user and producer accuracies reached 95.38% and 93.58%, respectively. The spatial distribution of the classification results showed excellent agreement with field observations, demonstrating the robustness of this method in salt marsh vegetation classification.

In summary, the classification results indicate that single data sources (optical or SAR) have limitations in distinguishing salt marsh vegetation types. However, the integration of multi-source data, including optical data, SAR polarization ratios, and polarization differences, leverages the complementary strengths of each feature set, enabling high-accuracy vegetation classification. This integrated approach not only demonstrates excellent stability and reliability in classifying different vegetation types but also provides critical support for the dynamic monitoring of complex ecosystems.

## 5. Discussion

**Figure 8:**
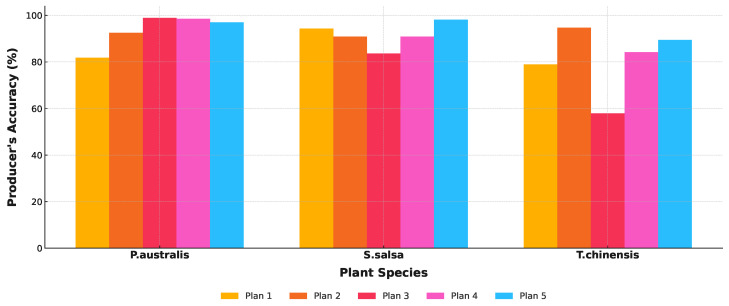
Producer’s accuracy bar chart.

**Figure 9:**
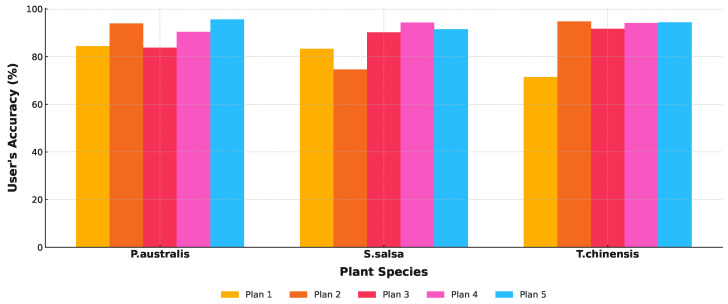
User’s accuracy bar chart.

Figure 8 and Figure 9 illustrate the classification results of three salt marsh vegetation types (*P. australis*, *S. salsa*, and *T. chinensis*) based on five different classification approaches (Plan 1 to Plan 5) with the left panel showing the study area and the right panels highlighting a detailed comparison of methods. Each plan integrates different combinations of Sentinel-2 optical data and Sentinel-1 SAR features, revealing progressive improvements in classification accuracy and ecological relevance.

Plan 1, which uses only Sentinel-2 optical data, demonstrates significant limitations. While *S. salsa* is reasonably identified in high-salinity areas, *T. chinensis* and *P. australis* exhibit substantial overlap, reflecting the inability of optical data alone to distinguish dense or spectrally similar vegetation types. Plan 2, based solely on Sentinel-1 SAR data, improves the delineation of *S. salsa*, particularly in tidal flats, by leveraging SAR’s sensitivity to structural and moisture variations. However, the overlap between *T. chinensis* and *P. australis* persists, as SAR lacks the phenological detail necessary for precise discrimination.

Plan 3 integrates Sentinel-2 data with SAR polarization ratios (VH/VV), improving classification performance. The inclusion of polarization ratios enhances the detection of structural differences, leading to more distinct boundaries for *S. salsa* and a partial reduction in overlap between *T. chinensis* and *P. australis*. Plan 4 incorporates SAR polarization differences (VV-VH) with Sentinel-2 data, further refining the spatial distribution of vegetation. Polarization differences effectively capture subtle variations in vegetation density and height, resulting in clearer separation between the three types.

Plan 5, which combines Sentinel-2 optical data with both SAR polarization ratios and differences, achieves the best classification accuracy. This comprehensive approach integrates spectral, structural, and phenological features, yielding highly distinct spatial boundaries and minimal overlap. *S. salsa* is accurately mapped in high-salinity zones near tidal flats, while *T. chinensis* and *P. australis* are clearly separated, particularly in transitional zones. The results align closely with field observations, confirming the ecological validity of this method.

**Figure 10:**
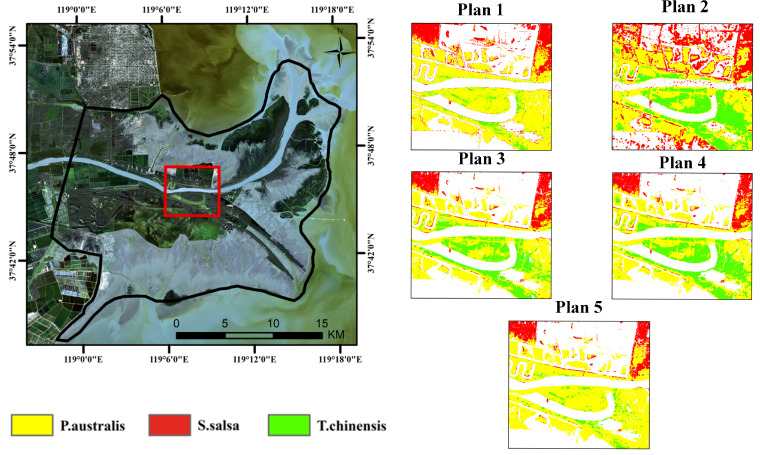
Detailed comparison of typical salt marsh vegetation classification.

Comparatively, Plans 1 and 2, relying on single-source data, exhibit substantial limitations in distinguishing vegetation types, particularly between *T. chinensis* and *P. australis*. Plans 3 and 4 show marked improvements through the integration of optical and SAR features, while Plan 5 demonstrates the superiority of combining complementary features for robust classification. The comparison in the detailed view of Figure 10 more clearly highlights the aforementioned points.

The ecological implications are significant. *S. salsa* dominates high-salinity tidal flats, reflecting its tolerance to extreme conditions. *T. chinensis* occupies mid-elevation zones, while *P. australis* thrives in lower-salinity inland areas. These patterns align with known ecological gradients in salt marshes, underscoring the utility of Plan 5 for accurate mapping and ecological monitoring.

In conclusion, the integration of Sentinel-2 and Sentinel-1 features, particularly in Plan 5, effectively overcomes the limitations of single-source data. This approach combines spectral and structural characteristics to achieve optimal accuracy, providing a reliable framework for salt marsh vegetation classification. The progressive improvement across plans highlights the value of multi-source data fusion, offering critical insights for conservation planning, ecological monitoring, and understanding the impacts of environmental changes on coastal ecosystems. They also provide useful structural information, further enhancing classification accuracy.

## 6. Conclusions

The main conclusions of this study are as follows. (1) The deep integration of optical phenological features (the start and end of the growing season, maximum NDVI values, and seasonal variation trends) and SAR polarization features (VV/VH ratio and VV-VH difference) significantly improved the classification accuracy of typical salt marsh vegetation in the Yellow River Delta. The overall classification accuracy reached 93.51%, with a Kappa coefficient of 0.917, validating the effectiveness of combining phenological and polarization features. (2) The integration of phenological and polarization features performed particularly well in classifying complex wetland environments, effectively addressing challenges associated with spectral overlap or structurally similar vegetation types, such as *P. australis* and *T. chinensis*, while also enhancing the recognition of salt-tolerant vegetation like *S. salsa*. (3) SAR polarization features demonstrated advantages in distinguishing vegetation structures with the VV/VH ratio and VV-VH difference being particularly effective for identifying dense vegetation. Meanwhile, phenological features captured by optical data played a critical role in monitoring vegetation dynamics and extracting salt-tolerant vegetation, such as *S. salsa*. The combination of these complementary datasets overcame the limitations of single-source methods.

The proposed multi-source data fusion method extends the potential applications of multi-temporal optical and SAR data in dynamic ecosystem monitoring, providing a reliable framework for wetland conservation and ecological restoration. Moreover, Sentinel-1 and Sentinel-2 data offer high temporal and spatial resolution and are freely available, making this approach highly applicable for wetland vegetation classification and ecosystem monitoring.

This method advances wetland vegetation classification and offers a robust framework for monitoring coastal ecosystems. The integration of structural and phenological data provides essential insights for wetland conservation, ecological restoration, and sustainable management. Future studies can further refine this approach by incorporating higher-resolution sensors or advanced algorithms to enhance classification accuracy and ecological monitoring capabilities.

## Figures and Tables

**Table 1:** Selection of remote sensing data sources.

Data	Jan.	Feb.	Mar.	Apr.	May	Jun.	Jul.	Aug.	Sep.	Oct.	Nov.	Dec.
Sentinel-1	06	11	06	11	05	10	23	09	02	03	01	02
	18	23	13	18	17	17		16	09	08	08	07
	30		18	23	24	22		21	14	15	13	19
			23		29			28	26	27	19	31
											20	
											25	
Sentinel-2	02	26	17	06	01	05	10	04	08	18	02	17
	12		22	11	06	20	15	19	18	23	07	22
	17		27	21	21		20	14	28		22	27
	22						25	29			25	
							30				27	

**Table 2:** Description and calculation of features.

Features	Feature Description	Calculation Method
Backscattering Characteristics	VV-VH 01-12, VH/VV 01-12	The VV and VH polarization intensities, as well as the VV-VH polarization difference and VH/VV polarization ratio, were calculated for each image. The monthly averages were then computed.
Phenological Parameters	Maximum Value (MV)	The maximum value of the fitting curve, which often occurs between late summer and early autumn.
	Base Value (BV)	The minimum value of the fitting curve, which often occurs in winter.
	Start of Season (SOS)	The day when the fitting curve reaches 50% of the difference between the BV and MV during the green-up period.
	End of Season (EOS)	The day when the fitting curve reaches 50% of the difference between the BV and MV during the senescence period.
	Rate of Increase (ROI)	The ratio of the difference in values ranging from 20% to 80% during the green-up season and the corresponding time difference.
	Rate of Decrease (ROD)	The ratio of the difference in values ranging from 20% to 80% during the senescence season and the corresponding time difference.

**Table 3:** Experimental scheme.

Plan	Feature Combinations
1	SOS, EOS, MV, BV, ROI, ROD
2	VH/VV, VV-VH
3	SOS, EOS, MV, BV, ROI, ROD, VH/VV
4	SOS, EOS, MV, BV, ROI, ROD, VV-VH
5	SOS, EOS, MV, BV, ROI, ROD, VV-VH, VH/VV

**Table 4:** Performance metrics of different plans.

Plan	Cross-Validation	Precision	Recall	F1-Score
1	0.8226 ± 0.0166	0.9206	0.8249	0.8418
2	0.9492 ± 0.0121	0.967	0.9607	0.9625
3	0.9509 ± 0.0168	0.9651	0.9609	0.9622
4	0.9601 ± 0.0134	0.9614	0.9543	0.9559
5	0.9616 ± 0.0145	0.9703	0.9603	0.9611

**Table 5:** Classification accuracy statistics.

Plant Species	S2		(VV-VH)+(VH/VV)		S2+(VH/VV)		S2+(VV-VH)		S2+(VV-VH)+(VH/VV)	
	**PA(%)**	**UA(%)**	**PA(%)**	**UA(%)**	**PA(%)**	**UA(%)**	**PA(%)**	**UA(%)**	**PA(%)**	**UA(%)**
*P. australis*	81.81	84.37	92.53	93.93	98.97	83.75	98.50	90.41	97.01	95.58
*S. salsa*	94.33	83.33	90.90	74.62	83.63	90.19	90.90	94.33	98.18	91.52
*T. chinensis*	78.94	71.42	94.73	94.73	57.89	91.66	84.21	94.11	89.47	94.44
Kappa	0.7208		0.8338		0.8391		0.8757		0.9172	
OA (%)	77.93		87.03		87.5		90.27		93.51	

PA: producer’s accuracy; UA: user accuracy; OA: overall accuracy.

## Data Availability

The data presented in this study are available on request from the corresponding author.
